# Predictive model for risk of gastric cancer using genetic variants from genome‐wide association studies and high‐evidence meta‐analysis

**DOI:** 10.1002/cam4.3354

**Published:** 2020-08-10

**Authors:** Lixin Qiu, Xiaofei Qu, Jing He, Lei Cheng, Ruoxin Zhang, Menghong Sun, Yajun Yang, Jiucun Wang, Mengyun Wang, Xiaodong Zhu, Weijian Guo

**Affiliations:** ^1^ Department of Medical Oncology Fudan University Shanghai Cancer Center Department of Oncology Shanghai Medical College Fudan University Shanghai China; ^2^ Cancer Institute Collaborative Innovation Center for Cancer Medicine Fudan University Shanghai Cancer Center Shanghai China; ^3^ Department of Pathology Fudan University Shanghai Cancer Center Shanghai China; ^4^ Ministry of Education Key Laboratory of Contemporary Anthropology and State Key Laboratory of Genetic Engineering School of Life Sciences Fudan University Shanghai China; ^5^ Fudan‐Taizhou Institute of Health Sciences Taizhou China

**Keywords:** gastric cancer, genome‐wide association study, predictive model, prognosis, susceptibility

## Abstract

Genome‐wide association studies (GWAS) have identified some single nucleotide polymorphisms (SNPs) associated with the risk of gastric cancer (GCa). However, currently, there is no published predictive model to assess the risk of GCa. In the present study, risk‐associated SNPs derived from GWAS and large meta‐analyses were selected to construct a predictive model to assess the risk of GCa. A total of 1115 GCa cases and 1172 controls from the eastern Chinese population were included. Logistic regression models were used to identify SNPs that correlated with the risk of GCa. A predictive model to assess the risk of GCa was established by receiver operating characteristic curve analysis. Multifactor dimensionality reduction (MDR) and classification and regression tree (CART) were applied to calculate the effect of high‐order gene‐environment interactions on risk of the cancer. A total of 42 SNPs were selected for further analysis. The results revealed that *ASH1L* rs80142782, *PKLR* rs3762272, *PRKAA1* rs13361707, *MUC1* rs4072037, *PSCA* rs2294008, and *PLCE1* rs2274223 polymorphisms were associated with a risk of GCa. The area under curve considering both genetic factors and BMI was 3.10% higher than that of BMI alone. MDR analysis revealed that rs13361707 and rs4072307 variants and BMI had interaction effects on susceptibility to GCa, with the highest predictive accuracy (61.23%) and cross‐validation consistency (100/100). CART analysis also supported this interaction model that non‐overweight status and a six SNP panel could synergistically increase the susceptibility to GCa. The six SNP panel for predicting the risk of GCa may provide new tools for prevention of the cancer based on GWAS and large meta‐analyses derived genetic variants.

## Graphical abstract

We identifed a six‐SNPs panel from GWAS and large meta‐analysis for predicting risk of gastric cancer, which may provide new tools for gastric cancer prevention.

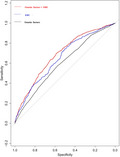

## 1 INTRODUCTION

Gastric cancer (GCa) is one of the leading causes of cancer‐related deaths worldwide and is the second most common malignancy after lung cancer in China. According to the statistics of China in 2015, there were approximately 679,100 new cases of GCa and 498,000 deaths, accounting for 15.8% of the cases and 17.7% of cancer deaths, respectively.^1^

As a heterogeneous disease characterized by epidemiology and histopathology, the mechanism underlying the etiology of GCa is not fully understood. It is well known that environmental factors, such as *Helicobacter pylori* (*Hp*) infection and dietary habits, play critical roles in increasing the risk of GCa.^2^, ^3^, ^4^ However, a disturbing aspect is that the risk of GCa is different even among people who are exposed to the same risk factors. For example, there is a high rate of *H pylori* infection worldwide (approximately 50%); however, only 1%‐2% of the total individuals will develop GCa in their lifetime, indicating that other factors can lead to increased risk of GCa.

Single nucleotide polymorphisms (SNPs), which were identified as minor allele frequencies of single nucleotides, observed in more than 1% of the general population, have been reported to be associated with both cancer predisposition and response to therapy.^5^, ^6^ Genome‐wide association studies (GWAS) have identified a series of germline alterations associated with the risk of lung,^7^ gastric,^8^ and prostate cancers,^9^ among others. The utility of genetic variants in early cancer prevention was also emphasized by some predictive models with sufficient ability to discriminate patients with different cancer risks.^10^ The majority of the SNPs associated with predisposition to GCa were derived from previous GWAS,^8^, ^11^, ^12^, ^13^ and were successfully reproduced by subsequent large case‐control studies. These SNPs, which particularly correlated with non‐cardia or cardia GCa, were also identified in a recent genome‐wide association study.^14^ Moreover, Shen et al identified potential new loci for non‐cardia gastric cancer by pooled analysis of two Chinese GWAS.^15^ Recently, a large meta‐analysis comprehensively reviewed genetic variants that predisposed an individual to GCa, and identified high‐evidence germline SNPs associated with a risk of acquiring the cancer.^16^ These results provided evidence‐based tools for early cancer screening. However, to the best of our knowledge, to date, there is no predictive model with sufficient discriminative ability for GCa.

GCa usually progresses rapidly without obvious symptoms, if not diagnosed at an early stage; therefore, identifying biomarkers would be helpful in preventing the cancer and is the focus of research worldwide. There is an urgent necessity to construct a predictive model with high discriminative ability for cancer risk based on the high‐evidence loci derived from GWAS and large meta‐analyses.

## 2 MATERIALS AND METHODS

### 2.1 SNP selection

Common risk‐associated SNPs, confirmed with a high level of evidence, were selected from GWAS^8^, ^12^, ^13^, ^14^ and a meta‐analysis.^16^ The inclusion criteria were as follows: 1, SNPs associated with risk of GCa; 2, SNPs proven to have a significant *P* value (ie, less than .05).

### 2.2 Study subjects

A total of 1115 unrelated ethnic Han Chinese patients with newly diagnosed and histopathologically confirmed primary GCa were recruited from Fudan University Shanghai Cancer (FUSCC) in Eastern China between January 2009 and March 2011. Patients with diseases other than histopathologically confirmed primary GCa were excluded. A total of 1172 age, sex, smoking, and drinking‐matched cancer‐free ethnic Han Chinese healthy controls were recruited from the Taizhou Longitudinal (TZL) study conducted during the same period in Eastern China. Blood samples of patients with GCa and cancer‐free controls were provided by the tissue bank of the FUSCC and the TZL study, respectively. All subjects provided written informed consent to donate their biological samples to the tissue bank for scientific research. Demographic data and environmental exposure history of each patient were collected. Clinical information of these patients was also collected. This research protocol was approved by the FUSCC Institutional Ethics Review Board.

### 2.3 Genotyping and quality control

DNA of the study subjects was extracted from peripheral blood. All the selected candidate SNPs were genotyped using a matrix‐assisted laser desorption/ionization time‐of‐flight (MALDI‐TOF) mass spectrometer using the MassARRAY Analyzer 4 platform (Sequenom, CA, USA). All primers were designed using the Assay Design Suite v2.0 from Mysequenom online software (www.mysequenom.com). The standard PCR was conducted in a total volume of 5 μL reaction system containing 10 ng of genomic DNA. One negative control and one duplicate control sample were used for quality control in every 96‐well plate. Genotyping results of 5% of the total patients were repeated, and the consistency was 100%.

### 2.4 Statistical methods

Genetic factors that correlated with the risk of GCa were calculated using unconditional logistic regression. The polygenic risk score (PRS) was calculated by the linear combination weighted by the coefficient derived from the stepwise logistic regression. To simulate the state of nature, frequency distribution based on the Hardy‐Weinberg equilibrium was also considered to calculate the PRS. The PRS was calculated using the following formula:

$$w_{n}=q^{2}+p^{2}OR_{n}+2pq{\text{OR}}_{n}$$

where *p* is the frequency of the risk allele, *q* is the frequency of the other allele, and OR is the odds ratio of the risk allele. *Wn* is the average PRS for our population, with respect to the corresponding *n*th SNP.

$$wPRS_{i}^{n}=\frac{{\text{OR}}_{n}^{\wedge }j}{W_{n}}$$

where $wPRS_{i}^{n}$ is the PRS for the nth SNP in the *i*th patient, and *j* is the dosage of risk allele the *i*th patient harbored.

Finally, the total PRS for the *i*th patient was calculated as follows:

$$wPRS_{i}=wPRS_{i}^{1}*wPRS_{i}^{2}*wPRS_{i}^{3}*\ldots \;\ldots wPRS_{i}^{n}$$

Specifically, a certain patient's PRS was calculated based on the genotype according to the candidate SNP and the weighted OR value. Subsequently, PRS was calculated as a continuous variable enrolling to the receiver operating characteristic (ROC) curve, and the predictive ability for the combined panel was displayed as area under curve (AUC). Bootstrapping tests were used to compare the AUCs. Classification and regression tree (CART) and multifactor dimensionality reduction (MDR) analyses were used to calculate the effect of high‐order gene‐environment interaction on the risk of GCa.

Of the total 2287 patients, there was missing data on BMI in 220 patients, and the data were filled by the random forests method, which has been demonstrated to be a high‐efficiency filling method in recent studies.^17^, ^18^

## 3 RESULTS

### 3.1 Candidate SNPs

Forty‐two SNPs were selected based on the criteria described above. The OR values of all 42 SNPs are included in Table S1, and the minor allele frequency of 29 SNPs in the Chinese population are included in Table S2. Additionally, the minor allele frequency of all 42 SNPs in our study patients are included in Table S3.

### 3.2 Population characteristics

An Eastern Chinese population of 1115 GCa patients and 1172 healthy controls were included in our study (Table 1). There was no statistically significant difference in the distribution of age, sex, smoking, and drinking status. BMI of healthy controls was higher than that of patients with GCa (*P < *.0001), indicating that BMI was a clinical factor in addition to genetic factors that affected the risk of the cancer. Clinical information from 926 patients was available for analysis. Of these patients, 444 had stage I‐II and 482 had stage III‐IV tumors. A total of 214 patients were diagnosed with mucinous adenocarcinoma or signet‐ring cell carcinoma, and 712 patients were diagnosed with adenocarcinoma. A total of 784 patients underwent surgery and 140 patients did not; 678 patients underwent chemotherapy, whereas 248 patients did not.

**Table 1:** Demographics of gastric cancer patients in this case‐control study within an eastern Chinese population

Variable	Case No. (100%)	Control No. (100%)	*P* value ^a^
All subjects	1115 (100.0)	1172 (100.0)	
Age (year)			.87
≤59	569 (51.0)	593(50.6)	
>59	546 (49.0)	579 (49.4)	
Sex			.61
Male	793 (71.1)	822 (70.1)	
Female	322 (28.9)	350 (29.9)	
Smoking			.49
Yes	677 (61.2)	734 (62.6)	
No	430 (38.8)	438 (37.4)	
Drinking			.66
Yes	261 (23.6)	267 (22.8)	
No	846 (76.4)	905 (77.2)	

^a^ *P* value for chi‐square test.

### 3.3 Predictive model for GCa risk based on GWAS‐derived genetic variations

**Figure 1:**
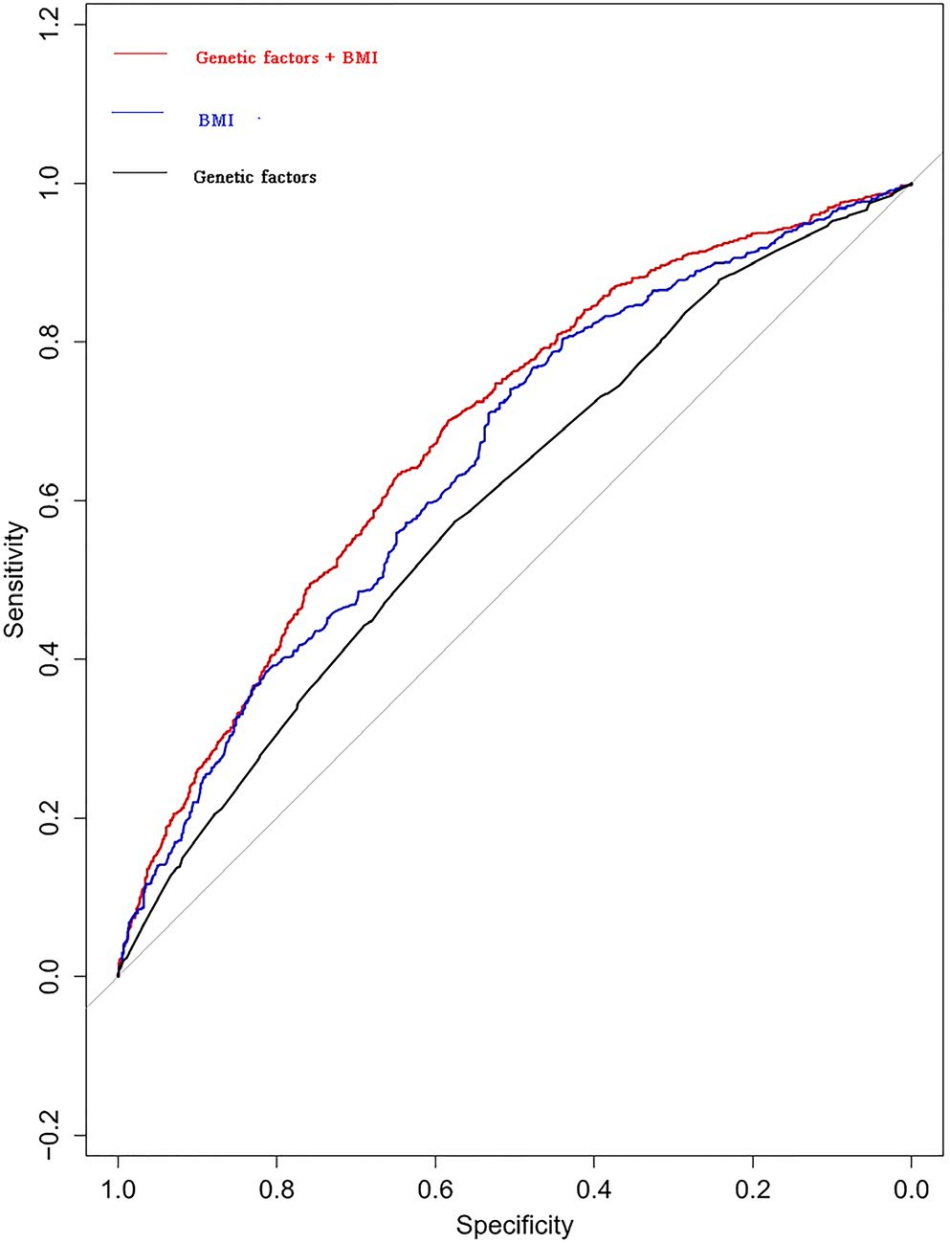
ROC curve assessing the predictive value of the panel of six SNPs associated with risk of GCa

Results of multivariate unconditional logistic regression indicated that rs13361707 C [C vs. T, OR = 1.47, 95% CI (1.30, 1.67), *P < *.0001], rs2294008 T [T vs. C, OR = 1.19, 95% CI (1.04, 1.36), *P = *.0108], rs4072037 T [T vs. C, OR = 1.38, 95% CI (1.16, 1.64), *P = *.0004], rs3762272 T [T vs. C, OR = 1.21, 95% CI (1.05, 1.39), *P = *.0082], rs2274223 G [G vs. A, OR = 1.35, 95% CI (1.16, 1.57), *P = *.0001], and rs80142782 T [T vs. C, OR = 1.36, 95% CI(1.07, 1.72), *P = *.0128] variants were predictors of increased risk of GCa (Table 2). A predictive model based on the ROC curve suggested that the AUC considering both BMI and genetic factors was significantly higher than that of genetic factors alone (AUC: 0.684 vs. 0.653, bootstrapping test, *P < *.0001), indicating that these SNPs were helpful, in addition to BMI, to discriminate an additional 3% of the patients with different risks for GCa (Figure 1).

**Table 2:** Genetic variants that was associated with increased gastric cancer risk

SNPs	Risk allele	variants	Case (%.)	Control (%.)	Crude OR (95%CI); *P*	^a^Adjusted OR (95%CI); *P*
rs13361707	C	CC	330 (29.89%)	249 (21.73%)	1.44 (1.28,1.63); <0.0001	1.47 (1.30,1.67); <0.0001
		CT	571 (51.72%)	576 (50.26%)		
		TT	203 (18.39%)	321 (28.01)		
rs2294008	T	CC	524 (47.46%)	615 (53.57%)	1.20 (1.05,1.36); 0.0070	1.19 (1.04,1.36); 0.0108
		CT	484 (43.84%)	446 (38.85%)		
		TT	96 (8.70%)	87 (7.58%)		
rs4072037	T	TT	840 (76.85%)	784 (68.53%)	1.42 (1.20,1.68); <0.0001	1.38 (1.16,1.64); 0.0004
		TC	233 (21.32%)	337 (29.46%)		
		CC	20 (1.83%)	23 (2.01%)		
rs3762272	T	TT	617 (55.94%)	589 (51.35%)	1.21 (1.06,1.38); 0.0057	1.21 (1.05,1.39); 0.0082
		TC	427 (38.71%)	467 (40.71%)		
		CC	59 (5.35%)	91 (7.93%)		
rs2274223	G	AA	636 (57.56%)	736 (64.71%)	1.31 (1.13,1.51); 0.0003	1.35 (1.16,1.57); 0.0001
		GA	409 (37.01%)	376 (32.75%)		
		GG	60 (5.43%)	36 (3.14%)		
rs80142782	T	TT	954 (88.17%)	972 (84.23%)	1.36 (1.08,1.71); 0.0095	1.36 (1.07,1.72); 0.0128
		CT	123 (11.37%)	176 (15.25%)		
		CC	5 (0.46%)	6 (0.52%)		

^a^ logistic regression model, adjusted for age, gender, BMI, smoking and drinking status.

### 3.4 Gene‐environment high‐order interaction

**Figure 2:**
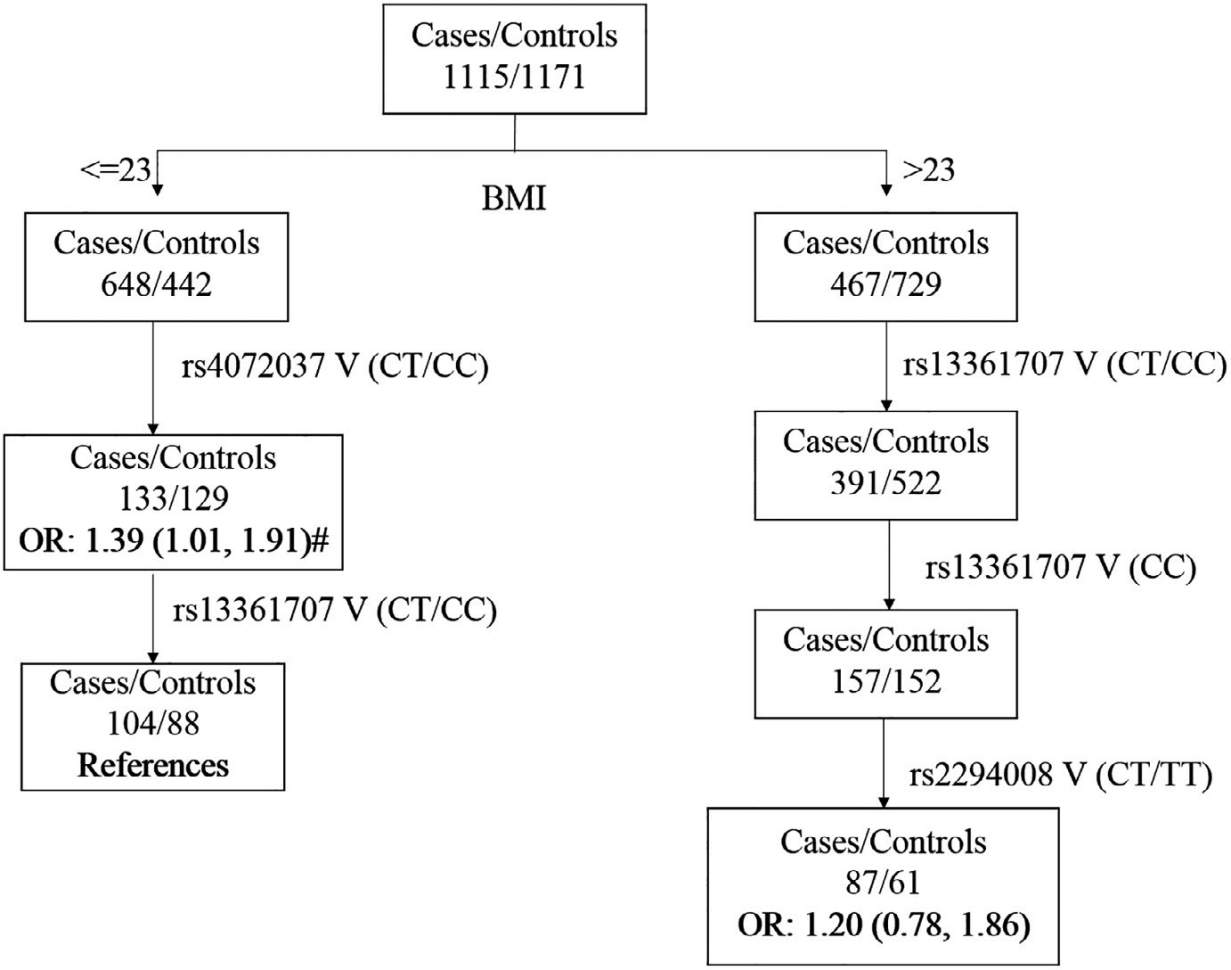
Classification and regression tree analysis of gene‐environment interaction on GCa risk. V, variants, #, *P* < .05

MDR analysis showed that the rs13361707 and rs4072307 variants and BMI had an interaction effect on susceptibility to GCa. This interaction presented the highest predictive accuracy (61.23%) and cross‐validation consistency (100/100) (Table 3). Similar to MDR, the results of CART analysis also indicated that BMI was the leading factor related to risk of GCa. Interestingly, CART analysis revealed a new interaction mode, which could be compared with the reference mode, wherein being non‐overweight (BMI < 23) and rs4072037 TT genotype could synergistically increase the risk of GCa by 39%[BMI < 23 and rs4072037 TT vs. reference mode, OR = 1.39, 95% CI (1.01, 1.91), *P = *.041] (Figure 2).

**Table 3:** MDR analysis for the prediction of gastric cancer risk

Number of risk factors	Best interaction models	Consistency of cross‐validation	Average of prediction errors	Permutation test (*P* value)
1	BMI	100/100	39.76%	<.0001
2	rs13361707, BMI	100/100	39.41%	<.0001
**3** ^a^	^a^ **rs13361707, rs4072037, BMI**	**100/100**	**38.77%**	**<.0001**
4	rs3762272, rs2274223, rs13361707, BMI	95/100	40.07%	<.0001

^a^ The best interaction model with minimal prediction error and highest consistency of cross‐validation was marked in bold.

## 4 DISCUSSION

GWAS have identified a number of genetic variants associated with the risk of GCa. For example, the first genome‐wide association study conducted in Japan identified *PSCA* rs2976392 as a susceptibility locus that correlated with the risk of diffuse GCa.^12^ A subsequent genome‐wide association study identified another polymorphism, *PLCE1* rs2274223, as a susceptibility germline SNP for cardia GCa.^11^ At the same time, the link between *PLCE1* rs2274223 SNP and cardia GCa was successfully reproduced by a genome‐wide association study in the Chinese population.^13^ Moreover, two SNPs, *PRKAA1* rs13361707 and *ZBTB20* rs9841504, which correlated with non‐cardia GCa were corroborated by another study in a Chinese population.^8^ However, there was lack of clarity whether these genetic variants contributed equally to the predisposition of GCa. Furthermore, to date, there are no PRS based studies which have included these genetic variants in the risk prediction of GCa. To the best of our knowledge, the present study is the first to construct a predictive model to assess the risk of GCa using well‐established SNPs derived from GWAS and high‐evidence based meta‐analyses. Importantly, our findings showed that these well‐established SNPs are helpful, in addition to clinical factors, to discriminate an additional 3% at‐risk population for GCa.

Gene‐environment interaction is another aspect that has been considered in assessment of the predisposition to GCa. Information about the interaction on the risk of GCa may be helpful for early cancer prevention in specific subsets. One large, prospective study performed in the Chinese population reported that low BMI correlated with an increased risk of GCa.^19^ However, to date, there is limited knowledge about the interaction between BMI and genetic factors and the susceptibility to GCa. In our study, individuals with low BMI (<23) carrying the risk alleles, rs13361707 C and rs4072037 T, were the most at‐risk population for GCa. In line with previous studies performed in Asian countries such as China,^20^ Japan,^21^ and Korea,^22^ our study also indicated that smoking habit did not have any effect in modifying the genetic risk for GCa. The interaction between *Hp* infection and the genetic risk for GCa was reported in a previous study with a limited sample size.^23^ Unfortunately, we could not elucidate the pattern of interaction due to lack of information about *Hp* infection.

The biological plausibility of the susceptibility loci found in our study can be reflected in their biological role in carcinogenesis. For example, as a susceptibility gene, *PRKAA1* encodes the catalytic α‐subunit of 5′ AMP‐activated protein kinase (AMPK), which plays an important role in cell energy consumption.^24^ A recent study reported that AMPK could activate autophagy and control cell proliferation by KDM2A‐dependent reduction of rRNA transcription.^25^ Moreover, AMPK can protect tumor cells from oxygen deficiency^26^ and promote its metastatic ability.^27^ A higher level of *PLCE1* expression was reported in tumor tissues than in normal tissues, and silencing the *PLCE1* gene in tumor cells could induce apoptosis.^28^ These observations support the role of the *PLCE1* gene in carcinogenesis. *MUC1*, as a master regulator of oncogenes, plays a vital role in cell proliferation, apoptosis resistance, and cell adhesion.^29^ Recently, a study revealed significantly higher expression of the *MUC1* protein in tumor cells than in normal cells through a specific cell ELISA technology, indicating that *MUC1* may play an important role in carcinogenesis.^30^ Another gene, *PKLR*, which was identified in our study, was also found to be a key regulator gene in carcinogenesis.^31^

The present study established a predictive model to assess the risk of GCa using high‐evidence genetic variants and detected the potential gene‐environment interaction, which may be helpful in prevention of the cancer. However, there are some limitations of this study. First, considering the retrospective nature of this study, the results must be validated by larger prospective studies. Second, the statistical power was largely reduced in the subgroup analysis due to small sample size.

## 5 CONCLUSIONS

The rs13361707 C, rs2294008 T, rs4072037 T, rs2274223 G, rs3762272 T, and rs80142782 T variants were associated with an increased risk of GCa. A predictive model based on these genetic variants showed substantial ability to discriminate additional at‐risk individuals. Gene‐environment interaction effects were detected on susceptibility to GCa among the rs13361707 and rs4072307 variants and BMI. Larger prospective studies are needed to validate our results.

## CONFLICT OF INTEREST

None declared.

## AUTHOR CONTRIBUTIONS

1. Conception and design: Lixin Qiu, Xiaofei Qu and Weijian Guo; 2. Administrative support: Weijian Guo, Xiaodong Zhu, Mengyun Wang; 3. Provision of study materials or patients: Lixin Qiu, Mengyun Wang and Weijian Guo; 4. Collection and assembly of data: Lixin Qiu, Xiaofei Qu, Jing He and Lei Cheng; Data analysis and interpretation: Xiaofei Qu, Jing He and Lei Cheng; 6. Manuscript writing: Lixin Qiu, Xiaofei Qu and Lei Cheng; 7. Final approval of manuscript: All authors.

## Supporting information

Table S1‐S3

## Data Availability

The data that support the findings of this study can be made available from the corresponding author upon reasonable request.
