# Supplementary material for: Predictive model for risk of gastric cancer using genetic variants from genome‐wide association studies and high‐evidence meta‐analysis
Source: Cancer Med. 2020 Aug 10;9(19):7310–6. doi: 10.1002/cam4.3354 (PMC7541133; doi:10.1002/cam4.3354)
Supplement: Supplementary file 1 — Table S1‐S3 [file CAM4-9-7310-s001.docx]

| **Supplementary table 1, The OR value of 42 Candidate SNPs in previous research** | | |
| --- | --- | --- |
| SNPs | OR (95% CI) | *P* value |
| rs139120256 | 1.35(1.03-1.77) | 2.70E-02 |
| rs79776715 | 1.38(1.10-1.72) | 4.51E-03 |
| rs17647701 | 1.19(1.06-1.33) | 2.63E-03 |
| rs7839487 | 1.25(1.08-1.44) | 2.63E-03 |
| rs76845414 | 0.75(0.63-0.91) | 2.42E-03 |
| rs78747266 | 1.44(1.15-1.81) | 1.73E-03 |
| rs78826154 | 1.44(1.15-1.81) | 1.52E-03 |
| rs77065565 | 1.22(1.08-1.38) | 1.46E-03 |
| rs61765798 | 1.18(1.07-1.31) | 1.27E-03 |
| rs9908993 | 0.89(0.83-0.95) | 7.52E-04 |
| rs187150116 | 1.50(1.19-1.88) | 4.83E-04 |
| rs12308015 | 1.13(1.06-1.21) | 3.29E-04 |
| rs2114910 | 1.13(1.06-1.20) | 3.09E-04 |
| rs77283376 | 1.27(1.12-1.43) | 2.10E-04 |
| rs12499763 | 1.18(1.08-1.28) | 1.43E-04 |
| rs74684850 | 0.75(0.65-0.87) | 1.28E-04 |
| rs13220767 | 1.92(1.37-2.67) | 1.25E-04 |
| rs75797460 | 0.81(0.72-0.90) | 1.04E-04 |
| rs532454 | 0.86(0.79-0.93) | 9.25E-05 |
| rs11754426 | 1.24(1.11-1.37) | 8.41E-05 |
| rs851911 | 1.27(1.13-1.43) | 6.49E-05 |
| rs72627187 | 0.87(0.81-0.93) | 3.35E-05 |
| rs431387 | 0.74(0.65-0.85) | 2.21E-05 |
| rs74402387 | 1.62(1.29-2.02) | 2.16E-05 |
| rs2294008 | 1.18(1.10-1.27) | 5.94E-06 |
| rs1108143 | 1.34(1.21-1.49) | 1.07E-08 |
| rs7712641 | 0.83(0.76-0.88) | 4.33E-09 |
| rs4072037 | 0.74(0.68-0.81) | 9.68E-12 |
| rs80142782 | 0.62(0.54-0.70) | 2.55E-13 |
| rs3087465 | 0.69(0.62-0.77) | 1.54E-11 |
| rs3762272 | 0.71(0.63-0.80) | 1.42E-08 |
| rs3834129 | 0.73(0.62-0.87) | 3.48E-04 |
| rs13361707 | 1.41(1.33-1.48) | <1.00E-20 |
| rs1695 | 1.19(1.09-1.30) | 0.0000788 |
| rs1799724 | 1.17(1.05-1.31) | 0.006 |
| rs2274223 | 1.57(1.49-1.65) | <1.00E-20 |
| rs2976392 | 0.81(0.75-0.87) | 1.42E-08 |
| rs8176719 | 1.26(1.16-1.29) | 1.92E-13 |
| rs9841504 | 0.58(0.67-0.72) | 6.70E-07 |
| rs13042395 | 0.80(0.71-0.88) | 6.90E-04 |
| rs10074991 | 0.83(0.89-0.88) | 4.83E-26 |
| rs2294693 | 1.14(1.09-1.20) | 7.22E-08 |

| **Supplementary Table 2**, 29 Candidate SNP effect allele frequency in Chinese population | | | | | |
| --- | --- | --- | --- | --- | --- |
| SNPs | | GROUP | | effect allele frequency (case, control) | |
| rs4072037 | | NCI | | 0.14,0.16 | |
| (T,C) | | Beijing | | 0.10,0.14 | |
|  | | Nanjing | | 0.13,0.16 | |
| rs80142782 | | NCI | | 0.06,0.07 | |
| (T,C) | | Beijing | | 0.03,0.07 | |
|  | | Nanjing | | 0.04,0.08 | |
| rs75797460 | | NCI | | 0.08,0.10 | |
| (G,A) | | Beijing | | 0.06,0.08 | |
|  | | Nanjing | | 0.05,0.08 | |
| rs61765798 | | NCI | | 0.13,0.10 | |
| (A,G) | | Beijing | | 0.11,0.11 | |
|  | | Nanjing | | 0.13,0.10 | |
| rs1108143 | | NCI | | 0.13,0.11 | |
| (A,G) | | Beijing | | 0.15,0.13 | |
|  | | Nanjing | | 0.15,0.12 | |
| rs78747266 | | NCI | | 0.03,0.02 | |
| (A,G) | | Beijing | | 0.04,0.03 | |
|  | | Nanjing | | 0.05,0.03 | |
| rs77283376 | | NCI | | 0.10,0.08 | |
| (G,T) | | Beijing | | 0.12,0.11 | |
|  | | Nanjing | | 0.12,0.11 | |
| rs139120256 | | NCI | | 0.02,0.01 | |
| (C,T) | | Beijing | | 0.02,0.02 | |
|  | | Nanjing | | 0.03,0.02 | |
| rs78826154 | | NCI | | 0.03,0.02 | |
| (C,G) | | Beijing | | 0.03,0.03 | |
|  | | Nanjing | | 0.03,0.02 | |
| rs12499763 | | NCI | | 0.20,0.16 | |
| (T,C) | | Beijing | | 0.17,0.17 | |
|  | | Nanjing | | 0.19,0.18 | |
| rs7712641 | | NCI | | 0.44,0.46 | |
| (T,C) | | Beijing | | 0.41,0.47 | |
|  | | Nanjing | | 0.45,0.48 | |
| rs13220767 | | NCI | | 0.01,0.01 | |
| (C,T) | | Beijing | | 0.05,0.03 | |
|  | | Nanjing | | 0.05,0.03 | |
| rs11754426 | | NCI | | 0.10,0.08 | |
| (T,C) | | Beijing | | 0.17,0.08 | |
|  | | Nanjing | | 0.14,0.09 | |
| rs187150116 | | NCI | | 0.04,0.03 | |
| (G,T) | | Beijing | | 0.02,0.03 | |
|  | | Nanjing | | 0.03,0.02 | |
| rs74402387 | | NCI | | 0.05,0.03 | |
| (T,G) | | Beijing | | 0.03,0.02 | |
|  | | Nanjing | | 0.03,0.02 | |
| rs76845414 | | NCI | | 0.03,0.04 | |
| (T,C) | | Beijing | | 0.02,0.03 | |
|  | | Nanjing | | 0.02,0.03 | |
| rs2294008 | | NCI | | 0.32,0.29 | |
| (C,T) | | Beijing | | 0.39,0.34 | |
|  | | Nanjing | | 0.36,0.32 | |
| rs7839487 | | NCI | | 0.05,0.04 | |
| (A,G) | | Beijing | | 0.12,0.04 | |
|  | | Nanjing | | 0.07,0.04 | |
| rs79776715 | | NCI | | 0.04,0.02 | |
| (T,C) | | Beijing | | 0.02,0.01 | |
|  | | Nanjing | | 0.02,0.01 | |
| rs77065565 | | NCI | | 0.10,0.09 | |
| (G,A) | | Beijing | | 0.10,0.09 | |
|  | | Nanjing | | 0.11,0.08 | |
| rs532454 | | NCI | | 0.75,0.79 | |
| (C,T) | | Beijing | | 0.75,0.77 | |
|  | | Nanjing | | 0.78,0.81 | |
| rs851911 | | NCI | | 0.07,0.06 | |
| (A,C) | | Beijing | | 0.13,0.07 | |
|  | | Nanjing | | 0.11,0.06 | |
| rs12308015 | | NCI | | 0.31,0.28 | |
| (T,C) | | Beijing | | 0.30,0.28 | |
|  | | Nanjing | | 0.31,0.27 | |
| rs2114910 | | NCI | | 0.55,0.51 | |
| (G,C) | | Beijing | | 0.55,0.54 | |
|  | | Nanjing | | 0.55,0.54 | |
| rs72627187 | | NCI | | 0.30,0.33 | |
| (T,C) | | Beijing | | 0.31,0.33 | |
|  | | Nanjing | | 0.29,0.33 | |
| rs17647701 | | NCI | | 0.07,0.07 | |
| (G,A) | | Beijing | | 0.15,0.07 | |
|  | | Nanjing | | 0.13,0.07 | |
| rs74684850 | | NCI | | 0.04,0.06 | |
| (A,G) | | Beijing | | 0.05,0.06 | |
|  | | Nanjing | | 0.04,0.06 | |
| rs9908993 | | NCI | | 0.49,0.53 | |
| (G,A) | | Beijing | | 0.49,0.52 | |
|  | | Nanjing | | 0.48,0.51 | |
| rs431387 | | NCI | | 0.93,0.94 | |
| (T,C) | | Beijing | | 0.89,0.96 | |
|  | | Nanjing | | 0.91,0.96 | |
| Abbreviation: NCI, national cancer institute. | | | |  | |

| **Supplementary Table 3**, 42 Candidate SNP effect allele frequency in Fudan University Shanghai Cancer Center gastric cancer patients and the control population | | | | | | |
| --- | --- | --- | --- | --- | --- | --- |
| SNP | Chr | Position | Alleles transform | MAF Cases | MAF Controls | HWE |
| rs3834129 | 2 | 201232809 | CTTACT>DEL | 0.214 | 0.194 | 0.140 |
| rs78747266 | 2 | 42725594 | A>G | 0.025 | 0.021 | 0.031 |
| rs1108143 | 2 | 234557214 | A>G | 0.098 | 0.098 | 0.339 |
| rs13361707 | 5 | 40791782 | C>T | 0.442 | 0.531 | 0.755 |
| rs2294008 | 8 | 142680513 | C>T | 0.306 | 0.270 | 0.622 |
| rs3087465 | 3 | 30605668 | G>A | 0.197 | 0.184 | 0.524 |
| rs4072037 | 1 | 155192276 | T>C | 0.125 | 0.167 | 0.055 |
| rs13042395 | 20 | 773867 | C>T | 0.373 | 0.387 | 0.743 |
| rs187150116 | 6 | 85691180 | G>T | 0.223 | 0.022 | 0.075 |
| rs11754426 | 6 | 79399426 | T>C | 0.968 | 0.088 | 0.941 |
| rs1695 | 11 | 67585218 | A>G | 0.188 | 0.195 | 0.396 |
| rs3762272 | 1 | 155291986 | T>C | 0.247 | 0.283 | 0.907 |
| rs2274223 | 10 | 94306584 | A>G | 0.239 | 0.195 | 0.148 |
| rs1799724 | 6 | 31574705 | C>T | 0.125 | 0.128 | 0.650 |
| rs75797460 | 1 | 197070213 | G>A | 0.191 | 0.101 | 0.954 |
| rs9841504 | 3 | 20938343 | C>G | 0.162 | 0.140 | 0.699 |
| rs7712641 | 5 | 89607147 | C>T | 0.474 | 0.468 | 0.604 |
| rs74402387 | 7 | 36962240 | T>G | 0.025 | 0.030 | 0.299 |
| rs78826154 | 3 | 164388560 | C>G | 0.027 | 0.030 | 0.970 |
| rs77065565 | 10 | 116257629 | G>A | 0.853 | 0.090 | 0.666 |
| rs12499763 | 4 | 83910247 | T>C | 0 | 0 |  |
| rs139120256 | 3 | 122948289 | C>T | 0.022 | 0.022 | 0.441 |
| rs532454 | 11 | 75443022 | T>C | 0.028 | 0.223 | 0.415 |
| rs851911 | 12 | 61940578 | A>C | 0.060 | 0.059 | 0.286 |
| rs2114910 | 12 | 100608337 | C>G | 0.486 | 0.474 | 0.882 |
| rs2294693 | 6 | 41037763 | T>C | 0.342 | 0.251 | 0.688 |
| rs2976392 | 8 | 142681514 | G>A | 0.300 | 0.270 | 0.458 |
| rs7839487 | 8 | 28608179 | A>G | 0.528 | 0.040 | 0.149 |
| rs8176719 | 9 | 133257521 | DEL>C | 0.429 | 0.458 | 0.615 |
| rs9908993 | 17 | 78637503 | A>G | 0.487 | 0.497 | 0.838 |
| rs10074991 | 5 | 40790449 | G>A | 0.442 | 0.536 | 0.956 |
| rs12308015 | 12 | 94894879 | T>C | 0.287 | 0.295 | 0.005 |
| rs13220767 | 6 | 64945266 | C>T | 0.002 | 0.004 | 0.882 |
| rs17647701 | 15 | 92597110 | G>A | 0.692 | 0.066 | 0.004 |
| rs61765798 | 1 | 18824073 | A>G | 0.113 | 0.114 | 0.247 |
| rs72627187 | 14 | 78138183 | T>C | 0.313 | 0.329 | 0.319 |
| rs74684850 | 16 | 78235875 | A>G | 0.049 | 0.055 | 0.394 |
| rs76845414 | 8 | 23042984 | T>C | 0.038 | 0.033 | 0.013 |
| rs77283376 | 2 | 43134421 | G>T | 0.091 | 0.087 | 0.931 |
| rs79776715 | 9 | 30386556 | T>C | 0.036 | 0.031 | 0.0001 |
| rs80142782 | 1 | 155515236 | T>C | 0.061 | 0.081 | 0.514 |
| rs431387 | 19 | 16178165 | C>T | 0.0009 | 0.0009 | 0.977 |
